# Supplementary material for: HIVprotI: an integrated web based platform for prediction and design of HIV proteins inhibitors
Source: J Cheminform. 2018 Mar 9;10:12. doi: 10.1186/s13321-018-0266-y (PMC5845081; doi:10.1186/s13321-018-0266-y)
Supplement: Supplementary file 2 — Additional file 2. Source code of HIVProtI web server. [file 13321_2018_266_MOESM2_ESM.zip › HIVprotI_Source-code/hivprotI/weka-3-6-6/doc/weka/classifiers/bayes/net/search/local/RepeatedHillClimber.html]

RepeatedHillClimber


---


|  |  |  |
| --- | --- | --- |
| **Overview** \| **Package** \| **Class** \| **Tree** \| **Deprecated** \| **Index** \| **Help** \| **Weka's home** | |  |
| **PREV CLASS**   **NEXT CLASS** | **FRAMES**    **NO FRAMES**     **All Classes** |  |
| SUMMARY: NESTED \| FIELD \| CONSTR \| METHOD | DETAIL: FIELD \| CONSTR \| METHOD |  |


---


## weka.classifiers.bayes.net.search.local Class RepeatedHillClimber

```
java.lang.Object
  weka.classifiers.bayes.net.search.SearchAlgorithm
      weka.classifiers.bayes.net.search.local.LocalScoreSearchAlgorithm
          weka.classifiers.bayes.net.search.local.HillClimber
              weka.classifiers.bayes.net.search.local.RepeatedHillClimber
```

**All Implemented Interfaces:**: java.io.Serializable, OptionHandler, RevisionHandler

---

``` public class RepeatedHillClimber extends HillClimber ```

This Bayes Network learning algorithm repeatedly uses hill climbing starting with a randomly generated network structure and return the best structure of the various runs.


Valid options are:

```
 -U <integer>
  Number of runs
```

```
 -A <seed>
  Random number seed
```

```
 -P <nr of parents>
  Maximum number of parents
```

```
 -R
  Use arc reversal operation.
  (default false)
```

```
 -N
  Initial structure is empty (instead of Naive Bayes)
```

```
 -mbc
  Applies a Markov Blanket correction to the network structure, 
  after a network structure is learned. This ensures that all 
  nodes in the network are part of the Markov blanket of the 
  classifier node.
```

```
 -S [BAYES|MDL|ENTROPY|AIC|CROSS_CLASSIC|CROSS_BAYES]
  Score type (BAYES, BDeu, MDL, ENTROPY and AIC)
```

**Version:**
:   $Revision: 1.6 $

**Author:**
:   Remco Bouckaert (rrb@xm.co.nz)

**See Also:**: Serialized Form

---

**Field Summary**

**Fields inherited from class weka.classifiers.bayes.net.search.local.LocalScoreSearchAlgorithm**

`TAGS_SCORE_TYPE`


**Constructor Summary**

`RepeatedHillClimber()`


| **Method Summary** | |
| --- | --- |
| `java.lang.String[]` | `getOptions()`             Gets the current settings of the search algorithm. |
| `java.lang.String` | `getRevision()`             Returns the revision string. |
| `int` | `getRuns()` |
| `int` | `getSeed()` |
| `java.lang.String` | `globalInfo()`             This will return a string describing the classifier. |
| `java.util.Enumeration` | `listOptions()`             Returns an enumeration describing the available options. |
| `java.lang.String` | `runsTipText()` |
| `java.lang.String` | `seedTipText()` |
| `void` | `setOptions(java.lang.String[] options)`             Parses a given list of options. |
| `void` | `setRuns(int nRuns)`             Sets the number of runs |
| `void` | `setSeed(int nSeed)`             Sets the random number seed |

**Methods inherited from class weka.classifiers.bayes.net.search.local.HillClimber**

`getInitAsNaiveBayes, getMaxNrOfParents, getUseArcReversal, setInitAsNaiveBayes, setMaxNrOfParents, setUseArcReversal, useArcReversalTipText`

**Methods inherited from class weka.classifiers.bayes.net.search.local.LocalScoreSearchAlgorithm**

`buildStructure, calcNodeScore, calcScoreWithExtraParent, calcScoreWithMissingParent, getMarkovBlanketClassifier, getScoreType, logScore, markovBlanketClassifierTipText, scoreTypeTipText, setMarkovBlanketClassifier, setScoreType`

**Methods inherited from class weka.classifiers.bayes.net.search.SearchAlgorithm**

`initAsNaiveBayesTipText, maxNrOfParentsTipText, toString`

**Methods inherited from class java.lang.Object**

`equals, getClass, hashCode, notify, notifyAll, wait, wait, wait`

**Constructor Detail**

### RepeatedHillClimber

```
public RepeatedHillClimber()
```


**Method Detail**

### getRuns

```
public int getRuns()
```

:   **Returns:**: number of runs

---


### setRuns

```
public void setRuns(int nRuns)
```

:   Sets the number of runs

    :   **Parameters:**: `nRuns` - The number of runs to set

---


### getSeed

```
public int getSeed()
```

:   **Returns:**: random number seed

---


### setSeed

```
public void setSeed(int nSeed)
```

:   Sets the random number seed

    :   **Parameters:**: `nSeed` - The number of the seed to set

---


### listOptions

```
public java.util.Enumeration listOptions()
```

:   Returns an enumeration describing the available options.

    :   **Specified by:**: `listOptions` in interface `OptionHandler` **Overrides:**: `listOptions` in class `HillClimber`
    :   **Returns:**: an enumeration of all the available options.

---


### setOptions

```
public void setOptions(java.lang.String[] options)
                throws java.lang.Exception
```

:   Parses a given list of options.


    Valid options are:

    ```
     -U <integer>
      Number of runs
    ```

    ```
     -A <seed>
      Random number seed
    ```

    ```
     -P <nr of parents>
      Maximum number of parents
    ```

    ```
     -R
      Use arc reversal operation.
      (default false)
    ```

    ```
     -N
      Initial structure is empty (instead of Naive Bayes)
    ```

    ```
     -mbc
      Applies a Markov Blanket correction to the network structure, 
      after a network structure is learned. This ensures that all 
      nodes in the network are part of the Markov blanket of the 
      classifier node.
    ```

    ```
     -S [BAYES|MDL|ENTROPY|AIC|CROSS_CLASSIC|CROSS_BAYES]
      Score type (BAYES, BDeu, MDL, ENTROPY and AIC)
    ```

    :   **Specified by:**: `setOptions` in interface `OptionHandler` **Overrides:**: `setOptions` in class `HillClimber`
    :   **Parameters:**: `options` - the list of options as an array of strings **Throws:**: `java.lang.Exception` - if an option is not supported

---


### getOptions

```
public java.lang.String[] getOptions()
```

:   Gets the current settings of the search algorithm.

    :   **Specified by:**: `getOptions` in interface `OptionHandler` **Overrides:**: `getOptions` in class `HillClimber`
    :   **Returns:**: an array of strings suitable for passing to setOptions

---


### globalInfo

```
public java.lang.String globalInfo()
```

:   This will return a string describing the classifier.

    :   **Overrides:**: `globalInfo` in class `HillClimber`
    :   **Returns:**: The string.

---


### runsTipText

```
public java.lang.String runsTipText()
```

:   **Returns:**: a string to describe the Runs option.

---


### seedTipText

```
public java.lang.String seedTipText()
```

:   **Returns:**: a string to describe the Seed option.

---


### getRevision

```
public java.lang.String getRevision()
```

:   Returns the revision string.

    :   **Specified by:**: `getRevision` in interface `RevisionHandler` **Overrides:**: `getRevision` in class `HillClimber`
    :   **Returns:**: the revision


---


|  |  |  |
| --- | --- | --- |
| **Overview** \| **Package** \| **Class** \| **Tree** \| **Deprecated** \| **Index** \| **Help** \| **Weka's home** | |  |
| **PREV CLASS**   **NEXT CLASS** | **FRAMES**    **NO FRAMES**     **All Classes** |  |
| SUMMARY: NESTED \| FIELD \| CONSTR \| METHOD | DETAIL: FIELD \| CONSTR \| METHOD |  |


---
